# Improving children’s visual health by integrating motor imagery training into physical education classes

**DOI:** 10.3389/fpsyg.2025.1587481

**Published:** 2025-06-09

**Authors:** Sheng Zhou, Yuanyuan Ren, Sien Ma, Meng Zhang, Rongbin Yin

**Affiliations:** ^1^Department of Basic Course, Suzhou City University, Suzhou, China; ^2^School of Physical Education, Soochow University, Suzhou, China

**Keywords:** motor imagery, visual health, uncorrected distance visual acuity, kinetic visual acuity, ciliary muscle, myopia, children

## Abstract

**Introduction:**

Myopia threatens healthy physical and mental development in children. Research suggests that motor imagery training could serve as a non-invasive and cost-effective non-pharmacological intervention to address myopia and promote health. Therefore, this study examined the effect of incorporating motor imagery training into physical education classes on children’s visual health.

**Methods:**

A 16-week intervention was conducted. The participants were 154 children divided into four groups: three experimental and one control. Group 1 performed motor imagery exercises with a visual target moving near and far, Group 2 performed physical activity imagery exercises combined with visual tasks, and Group 3 performed physical activity combined with visual tasks.

**Results:**

After the intervention, kinetic visual acuity (*p <* 0.05), accommodation facility (*p <* 0.01), and uncorrected distance visual acuity (*p <* 0.01) improved significantly in all experimental groups. Moreover, Groups 1 and 2 showed significant improvements in cognitive specific motor imagery abilities (*p <* 0.05). There were significant differences in kinetic visual acuity (*F* = 2.994, *p* = 0.033, *η*^2^ = 0.056), accommodation facility (*F* = 8.533, *p* < 0.001, *η*^2^ = 0.146), right-eye uncorrected distance visual acuity (*F* = 5.550, *p* = 0.001, *η*^2^ = 0.100) and left-eye uncorrected distance visual acuity (*F* = 2.667, *p* = 0.050, *η*^2^ = 0.051) among the four groups.

**Conclusion:**

Incorporating motor imagery training into physical education classes can improve children’s visual health by enhancing cognitive and visual skills. The findings of this study may help develop interventions to prevent myopia through activation of ciliary muscles.

## 1 Introduction

In a world full of visual information, the eyes are the window through which humans understand the external world. Eye health is an important component of universal health coverage (Burton et al., 2019). Vision plays an important role at every stage of human life. Eye health problems reduce individuals’ quality of life, limit access to education and work opportunities, and increase the economic burden on families and society. Therefore, proactive action focusing on eye health is required (Burton et al., 2021). Children’s eye health problems, particularly myopia, which hinders healthy physical and mental development in children and adolescents, are a significant concern. The risk of myopia development is directly increased by several objective factors, such as reduced exposure to the outdoors (Migneron-Foisy et al., 2017), reduced physical activity time (Jones et al., 2007), increased use of electronic products (Foreman et al., 2021), and increased close work and sedentary time (Harrington et al., 2019). Prevention and management of myopia among children are a priority in China, and effective interventions are urgently needed to reverse the deterioration of vision among children.

Research has shown that reduced accommodation is an early sign of myopia (Allen and O'Leary, 2006). According to accommodation theory, visual accommodation is primarily achieved through the contraction and relaxation of the ciliary muscle, thereby altering the shape of the lens. The ciliary muscle is a unique smooth muscle that exhibits certain characteristics of striated muscle and is innervated by both the sympathetic and parasympathetic nervous systems. Abnormalities in the structure, innervation, and function of the ciliary muscle are closely related to the onset and progression of myopia. Improving the accommodative function of the ciliary muscle is a key factor in enhancing children’s visual health.

In recent years, motor imagery training has been widely used for therapeutic, rehabilitation, and health promotion purposes (Yuan et al., 2021; Zhai et al., 2012). Imagery refers to the mental process of reproducing or reconstructing the body’s visual, auditory, tactile, and proprioceptive experiences in the mind without the direct involvement of external stimuli (Ji et al., 2016). Based on the psychoneuromuscular theory, imagery theory, and theories related to motor imagery and the autonomic nervous system (Song, 2015), motor imagery training has been shown to elicit neural activation of skeletal (striated) muscles, modulate autonomic nervous system activity, and induce changes in smooth muscles. Furthermore, imagery and perception share similarities in structure, function, and neural mechanisms; they interact with each other, and perception can be improved through appropriate imagery. Based on these theoretical foundations, this study considers that a motor imagery intervention targeting children’s vision is theoretically reasonable and feasible.

Based on the above, this study aims to experimentally examine the effects of motor imagery training, designed in accordance with the principle of ciliary muscle accommodation, on children’s visual acuity and to evaluate the differential effects of various intervention modalities. The intervention approaches included: (1) physical activity combined with visual tasks, (2) imagery training consisting of visual target movement exercises, and (3) imagery training consisting of physical activity exercises that integrate visual tasks. The effects of these different intervention modalities on children’s visual function were evaluated using three indicators: uncorrected distance visual acuity (UDVA), kinetic visual acuity (KVA), and accommodative facility.

The following hypotheses are proposed in this study: (1) Motor imagery training can effectively improve children’s UDVA, KVA, and accommodative facility; (2) Different motor imagery intervention protocols exert differential effects on children’s UDVA, KVA, accommodative facility, and imagery ability; (3) The imagery training consisting of visual target movement exercises is the most effective.

## 2 Materials and methods

### 2.1 Participants

G*Power 3.1 was used to determine the required sample size for a paired-sample *t*-test. Based on previous studies (Zhou et al., 2023), the effect size was set at 0.23, and input 0.05 for “*α* err prob” and 0.8 for “power (1-*β* err prob), the calculated total sample size was 151 participants. Consequently, we recruited 187 fourth-grade students, aged 9–10 years, from the Suzhou Science and Technology City Experimental Primary School.

The inclusion criteria were (1) UDVA or best corrected visual acuity ≥ 4.0; (2) No impairment of cognitive function and motor function, and ability to follow verbal instructions to complete exercises and test tasks.

The exclusion criteria were (1) diagnosed with ocular diseases that affect vision, such as congenital hyperopia, amblyopia, strabismus, etc. History of intraocular surgery or eye trauma; current use of orthokeratology lenses; or undergoing myopia correction treatment in hospitals or ophthalmic clinics; (2) diagnosed with mental disorders, cognitive impairment, physical disabilities, recent limb injuries, or history of surgery; (3) failure to complete the KVA and accommodative facility test; (4) submission of an incomplete questionnaire (one or more unanswered questions were considered invalid) or a questionnaire with most answers identical or following an obvious response pattern; (5) Transferring to another school or failure to meet the experiment requirements.

Considering the practical setting, we adopted the cluster random sampling method to select the participants. Specifically, among 12 intact classes (approximately 40 students per class) from the same school, we randomly selected 4 classes using a lottery method. The detailed procedure was as follows: the classes were numbered from 1 to 12, and each number was written on cards that were identical in appearance, texture, and size. These cards were placed into an opaque container, thoroughly mixed, and drawn without replacement by a person not involved in the intervention. The classes corresponding to the drawn numbers were selected as the study sample. Subsequently, each selected class was treated as a sampling unit and was randomly allocated to either an intervention group or the control group through another round of lottery drawing. The final sample comprised 154 participants divided into four group: Group 1 (*n* = 37), Group 2 (*n* = 40), Group 3 (*n* = 40), and the Control Group (*n* = 37). Baseline visual acuity indicators of the study participants are presented in Table 1.

**Table 1:** Participants’ baseline visual acuity indicators.

Group (*N*)	UDVA (left)	UDVA (right)	KVA	Accommodative facility
Group 1 (37)	4.811 ± 0.317	4.841 ± 0.368	0.490 ± 0.234	7.095 ± 2.140
Group 2 (40)	4.805 ± 0.323	4.857 ± 0.319	0.553 ± 0.317	6.550 ± 2.449
Group 3 (40)	4.837 ± 0.287	4.857 ± 0.262	0.484 ± 0.275	6.775 ± 2.178
Control Group (37)	4.795 ± 0.370	4.768 ± 0.400	0.451 ± 0.296	6.351 ± 1.996
*F*	0.124	0.603	0.868	0.782
*p*	0.946	0.614	0.459	0.506

All measurements and experiments were conducted within the school physical education curriculum.

### 2.2 Procedure and experimental manipulations

#### 2.2.1 Experimental scheme

The principle of imagery training is creating a situation to induce a state in the participant, using imagery to obtain an experience of a mental process, and ultimately, obtaining a new understanding of the exercise content and improving the corresponding ability. According to the imagery training approach developed by Simmons et al. (2008), participants in this study underwent motor imagery encoding prior to the formal imagery training. This required the students to physically perform the exercise content (involving either physical activities combined with visual tasks or observation of target movement exercises) in order to construct a mental representation of the correct exercise process in the brain. Conducting imagery training after actual practice helps achieve an optimal level of arousal. During the imagery encoding phase, the more accurate and robust the children’s memory of the actual exercise execution, the more effective their motor imagery practice is expected to be. After the encoding period, participants entered the motor imagery training phase, during which they performed imagery based on guided instructions without physically executing the movements. We used the following imagery training designs.

**Figure 1:**
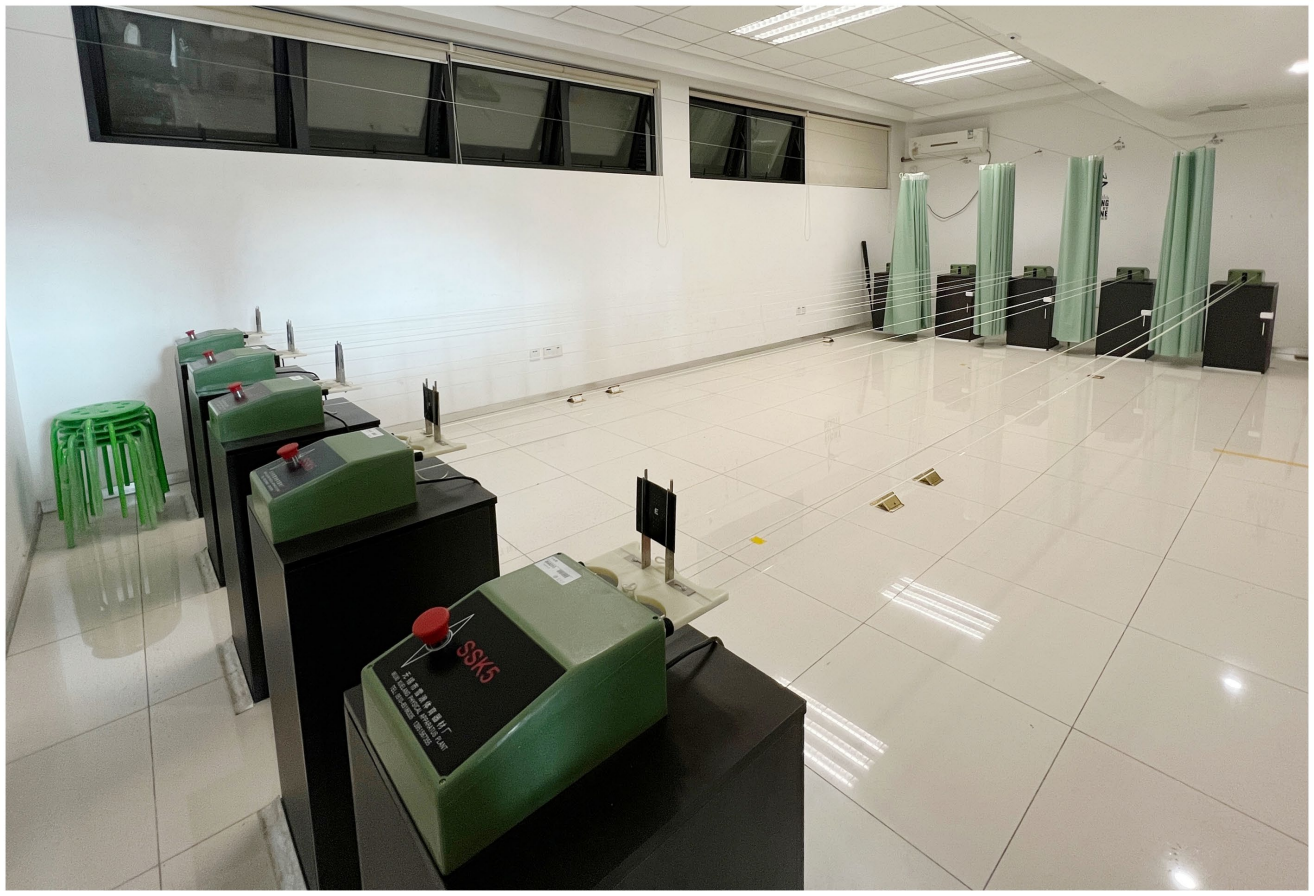
SSK-5 six-meter moving target.

For Group 1, the imagery training consisted of visual target movement exercises. During the encoding period, participants observed the far and near movement of the visual target using the SSK-5 six-meter moving target (Wuxi Xuelang Sports Equipment Factory), which fully embodied kinetic vision characteristics. The moving target equipment was placed in the school’s equipment room, with a six-meter moving track, letters, and patterns printed on the visual target, which were moved at a speed of 2 m/s (Figure 1). After the participants pressed the red button, the moving target in initial position started to move from far and near. The participants used both eyes to capture the movement of the track and size and content of the visual target, stopping at the end. Subsequently, the participants pressed the red button again, and the visual target moved from near to far back to the initial position. The participants used both eyes to track the visual target.

For Group 2, the imagery training consisted of physical activity exercises that incorporated visual tasks. The encoding training consisted of performing physical activities that incorporated visual tasks (mainly ball sports skill programs, including basketball dribbling, passing and catching, and football kicking inside and out). The training content of the encoding period was equal to that of Group 3. This combination likely imposed a higher cognitive load due to the simultaneous processing of motor and visual information.

The content of the motor imagery training in Group 1 and Group 2 focused on the visual change from not seeing to seeing during near and far vision. The intervention was performed three times a week, including one coding exercise and two imagery exercises. Each coding exercise was completed 30 times, and each successful recognition of the far and near visual target was considered to be the completion of one exercise frequency. The time value of the visual target presentation was used for 3 s. Imagery exercises program: 30 s of relaxation → 1 min of attention → 3 min of imagery exercises → 30 s of relaxation. Students followed the same frequency and time values as those of the actual exercise during the motor imagery exercise.

For Group 3, the intervention consisted of physical activity combined with visual tasks. The physical activity program with additional visual tasks was designed according to the Physical Education and Health Curriculum Implementation Plan for Compulsory Education in Jiangsu Province (Ministry of Education, 2022). The exercises were mainly based on ball sports skills, including basketball dribbling, passing and catching, football kicking, and dribbling. Considering safety, effectiveness, fun, and gradual progression, the study reasonably attached kinetic visual tasks to the exercises and added appropriate visual aids, ensuring that the visual targets could be used throughout the movement exercises and that the students could actively capture the visual targets with both eyes during the exercises. The sizes of the visual targets were set according to a standard logarithmic visual acuity chart. Exercise required a reasonable frequency and time to maximize its effect. Based on the results of a previous study (Yin et al., 2022; Zhou et al., 2023), Group 3 completed the intervention three times per week, and each intervention required the completion of 30 frequency exercises; each successful recognition of the distance and near vision targets was regarded as one practice frequency completed. The time value for the presentation of the visual target was 3 s. The teaching content is shown in Table 2.

**Table 2:** Teaching content.

Category	Sports event	Content	Visual target and equipment	Training requirements
Basic activity skills	Running	Fast running, shuttle running, timed running, obstacle running, relay running, and other related exercises	Sandbags, softballs, solid balls, basketballs, footballs, volleyballs, sign barrels, sight frames, ladders, number cards, letter cards, word cards, etc.	Visually capturing numbers, words, or letters on the visual targets during exercises
	Jumping	Exercises related to standing long jump, squatting long jump, and leaping high jump		
	Throwing	Exercises related to running pitching softballs (sandbags) and pushing solid balls		
Ball sports	Basketball	Comprehensive drills and competitions in dribbling moving, passing and receiving, dribbling in different directions, shooting from spots, etc.		
	Football	Practice and competitions in inner and outer instep, catching, and back-front kicking, dribbling, etc.		
	Badminton	Combination exercises and teaching games such as moving footwork, moving front two-handed padding, side underhand serves, etc.		

During the intervention period, participants in the control group continued their regular school activities without receiving any additional intervention. They attended standard physical education classes and participated in routine classroom lessons, but did not engage in any vision-related exercises or imagery training.

**Figure 2:**
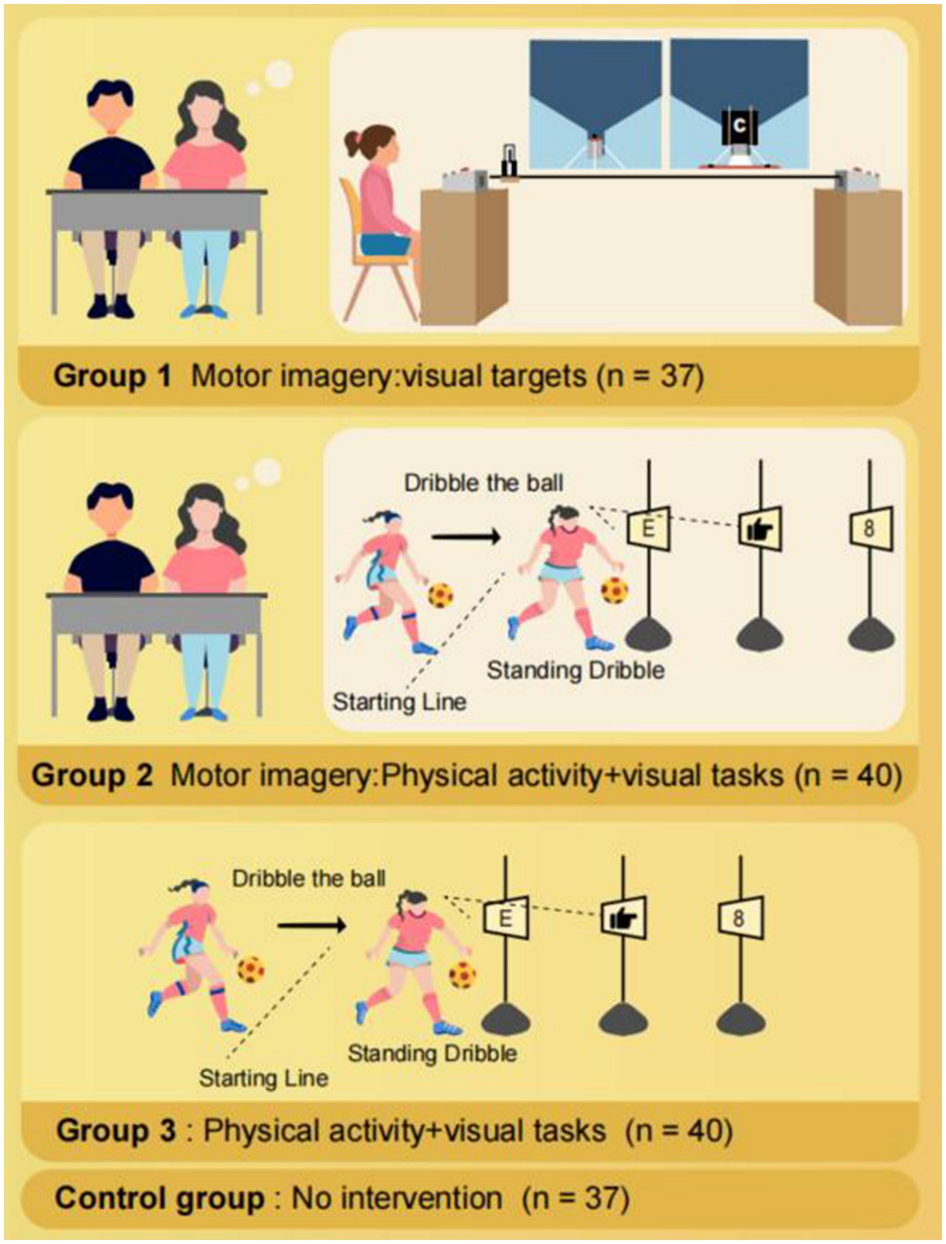
Experimental scheme diagram.

The participants, instructors, and evaluators were blinded to the allocation of the groups. They were not informed of which experimental or control group they were assigned to, nor of the specific hypotheses associated with each group. All interventions in the experimental group were conducted as part of the school physical program. During the study period, we maintained regular communication with both the participants and their parents to monitor the students’ daily activities, including weekend physical activity levels and visual behaviors, etc., in order to prevent significant lifestyle changes that might affect the validity and reliability of the study results. The experimental scheme is presented in Figure 2.

#### 2.2.2 Motor imagery training objectives

The effect of motor imagery training is closely related to the content of imagery training, and differences exist in the use of different types of motor imagery (Farah et al., 1988). To improve specific abilities, effectively activate the corresponding parts or functions, and maximize the benefits of imagery training, the construction of a motor imagery training program needs to consider the actual characteristics of the task and develop targeted imagery training guidelines and imagery content (You et al., 2020). Therefore, this study used the Sport Imagery Questionnaire (SIQ) and UDVA test to analyze the imagery differences in children with different visual acuity levels and to identify the imagery characteristics of children with better visual acuity. This provided a basis for formulating targeted and effective voice guidance and the content of imagery training and improved the effect of subsequent imagery training.

The students participating in the test were fourth-grade students (aged 9–10 years) from the Suzhou Science and Technology City Experimental Primary School, divided into myopic and non-myopic groups with UDVA of 5.0 as the boundary (National Health Commission, 2021). The UDVA index and questionnaire scores showed that compared to myopic children, orthotopic children had higher scores on specific imagery, and the difference was statistically significant (*p* < 0.05). These results suggest that the improved visual acuity may be related to special cognitive imagery. Improving cognitive specific imagery could help manage myopia in children.

#### 2.2.3 Motor imagery training voice guidance

The motor imagery training prompts were designed with reference to the entries of the cognitive-specific imagery dimensions and were based on the description of realistic visual tasks to ensure positive guidance without aggravating the psychological burden of the participants.

For instance, motor imagery training in football practice combined with visual tasks used the following prompt:

(1) Slowly close your eyes, imagine that you walk out of the classroom, look up to the turquoise blue sky, a few white clouds floating, the air is filled with the fragrance of the flowers, and feel very relaxed. (2) You come to the football field, stand face-to-face with your partner six meters away, and put the football in the specified position. Listen to commands for football practice. (3) Prepare, gaze at the football, kick the ball, watch the ball move toward the far side, and stop moving. (4) Prepare, gaze at the football, partner kicks the ball, watch the ball move closer and stop moving. (5) Prepare, gaze at the ball, kick, stop, prepare, gaze at the ball, partner kick, stop (repeat 29 times). The practice is over, and you slowly walk out of the football field, look at the birds in the sky, and then look at the green meadow, take a deep breath, and the warm wind is blowing on your face, feeling very relaxed.

### 2.3 Outcome measures

#### 2.3.1 Uncorrected distance visual acuity

An international standard logarithmic visual acuity chart (GB11533-2011) was used for the UDVA test. The test method and process were performed in strict accordance with the standard in which the right eye was measured first, followed by the left eye. If the participants wore spectacles, they were asked to remove the spectacles for a short rest before measuring the UDVA. Participants stood at the marking line 5 m from the visual acuity meter and were required to say or point out the direction of the reticle pointed at by the tester within a short period of time. The value displayed on the line in which the last reticle was located with the correct answer was considered the participant’s visual acuity in that eye. The same researcher recorded the UDVA data of all participants.

#### 2.3.2 Kinetic visual acuity

The KVA was measured using the Kinetic Visual Acuity Tester (Shanghai Camelot Automation Technology Co., Ltd.), with KVA scores ranging from 0.1 to 1.6, with higher scores representing better levels of KVA. Each participant was tested three times at intervals of 30 s, and the average value was taken as the KVA score. The participants sat in front of the instrument with their upper body upright and eyes close to the eye aperture, looking inward. At the beginning of the test, a Landolt ring approaching 50 m away appeared in the apparatus with four notch directions: up, down, left, and right, and a simulated approach speed of 30 km/h. The participants used their dominant hand to hold the rocker and view the Landolt ring. The participants held the rocker in their dominant hand, saw the direction of the Landolt ring, and quickly pulled the rocker in the corresponding direction to complete the test. The same researcher measured all participants’ KVA using unified instructions and evaluation criteria.

#### 2.3.3 Accommodative facility

Accommodative facility tests were conducted strictly following the standard using ±2.00D Accommodative Flippers, which are made up of two pairs of lenses, one for distance vision (+2.00D) and the other for near vision (−2.00D), which can be adjusted according to the distance between the eyes of the participants. Each participant was seated in front of a desk, and a word rock card (20/30) was placed 40 cm in front of the participant’s eyes. The card contained 40 letters in 5-point font, which participants needed to identify in order from left to right during the test. A stopwatch was held in the tester’s hand to time the test (1 min), and the participant held a handle with their right hand and placed the flipper lens horizontally in front of both eyes. At the beginning of the test, the participants identified the first English letter on the card 40 cm in front of their eyes through the +2.00D lenses. The participant identified and named the letter that was blurred at first, then rotated the handle, identified the second English letter on the card with the −2.00D lenses, and clearly named the letter, completing a cycle. The change from a positive lens degree to a negative lens degree and back to a positive lens degree is called 1 cycle. The number of cycles in 1 min was measured in cycles per minute (cpm), with higher cpm values indicating a better accommodative facility.

#### 2.3.4 Sport imagery questionnaire

Based on motor imagery training objectives, the questions of the cognitive general imagery (CG) dimension and the cognitive-specific imagery (CS) dimension subscales of the SIQ questionnaire were selected and modified according to the characteristics of imagery training and visual target tracking to make them suitable for this study. Each subscale has six items, and each item is scored as 1, 2, 3, 4 or 5 points. The sum of the scores of all the questions in each subscale is the final score, which ranges from six to 30. Higher scores indicate greater use of such imagery. The reliability and validity tests showed that the internal consistency coefficients of the two subscales were 0.720 and 0.691, the correlation coefficient between the two dimensions was 0.591, and the overall internal consistency coefficient was 0.808, indicating that the questionnaire had good reliability. The results of the exploratory factor analysis (KMO value of 0.793) showed that the factor structure of the questionnaire was clear and had good construct validity.

### 2.4 Ethical considerations

This study was approved by the Ethics Committee of Soochow University (No. SUA20201010H01). All the procedures complied with the ethical standards of the Declaration of Helsinki, legal requirements, and international standards.

### 2.5 Statistical analyses

Data were statistically analyzed using SPSS26.0. The study utilized a data sample of 154 participants, which is considered a large sample. According to the Lindeberg-Lévy Central Limit Theorem, in large samples (*n* > 30), the sampling distribution tends to be normal, regardless of the shape of the data (Elliott and Woodward, 2007; Field, 2009). The changes in the index data of the four groups before and after the experiment were analyzed using a paired-sample *t*-test. The differences among the data of the groups were compared by one-way ANOVA and *post hoc* test, and the *t*-test and *F*-test were used to calculate the effect sizes by using Cohen’s *d* and *η*^2^, respectively. *η*^2^ < 0.06 was a small effect, 0.06 ≤ *η*^2^ ≤ 0.14 was a medium effect, and *η*^2^ > 0.14 was a large effect, 0.2 ≤ Cohen’s *d* < 0.5 was a small effect, 0.5 ≤ Cohen’s *d* < 0.8 was a medium effect, and Cohen’s *d* ≥ 0.8 was a large effect. The significance level of *α* = 0.05.

## 3 Results

### 3.1 Comparison of KVA between groups

**Figure 3:**
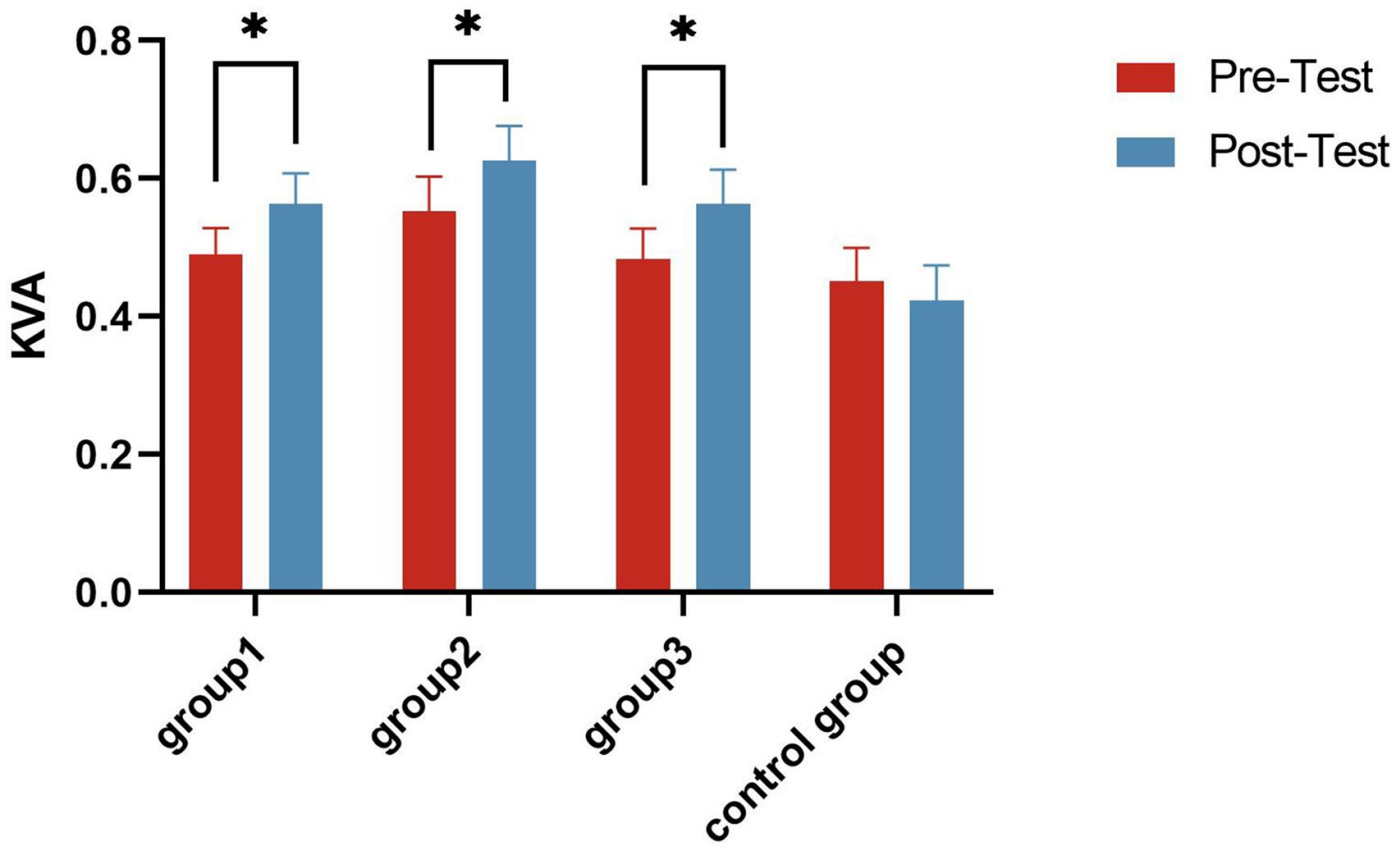
Kinetic visual acuity (KVA) change trend in groups. *p ≤ 0.05.

**Figure 4:**
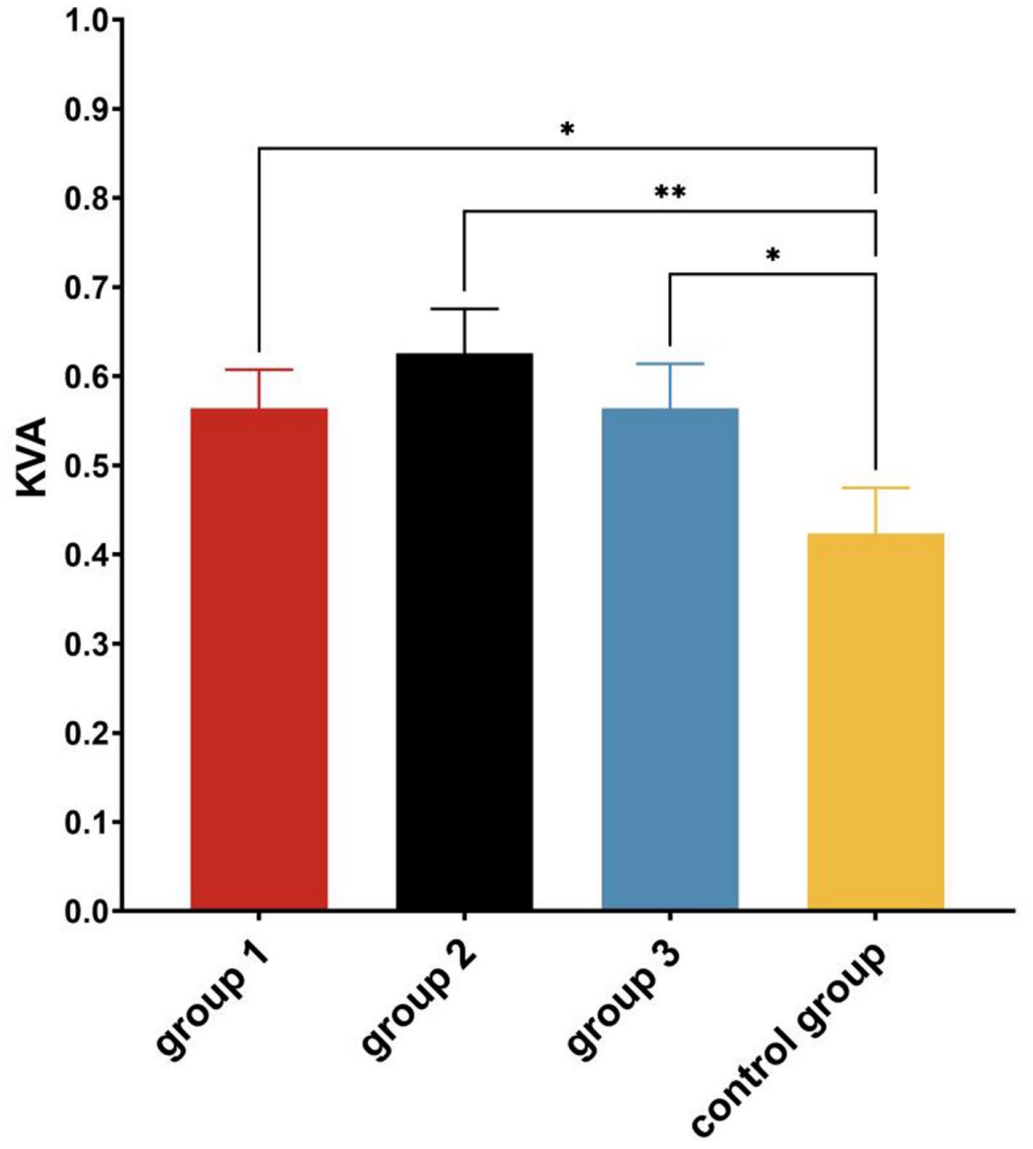
*Post hoc* multiple comparisons of KVA among groups after the experiment. *p ≤ 0.05; **p ≤ 0.01.

Paired samples *t*-tests showed that the KVA significantly increased in all experimental groups (*p* < 0.05) after the intervention (Table 3 and Figure 3). A one-way ANOVA showed that there were significant differences in KVA among the four groups (*F* = 2.994, *p* = 0.033, *η*^2^ = 0.056). *Post hoc* multiple comparisons showed that all three experimental groups had significantly better KVA than the Control Group (*p* < 0.05) (Figure 4).

**Table 3:** Comparison of KVA between the groups.

Group	Pre-test	Post-test	*t*/*(I-J)*	*P*	Cohen’s *d*
Group 1	0.490 ± 0.234	0.564 ± 0.265	−2.200	0.034	0.361
Group 2	0.553 ± 0.317	0.626 ± 0.317	−2.157	0.037	0.341
Group 3	0.484 ± 0.275	0.563 ± 0.318	−2.426	0.020	0.384
Control group	0.451 ± 0.296	0.423 ± 0.314	0.876	0.387	0.144
*F*	0.868	2.994			
*P*	0.459	0.033			
*η* ^2^	0.017	0.056			
*Post hoc*		Group 1 > Control group	0.140	0.049	0.461
		Group 2 > Control group	0.202	0.004	0.663
		Group 3 > Control group	0.140	0.045	0.460

### 3.2 Comparison of accommodative facility between groups

**Figure 5:**
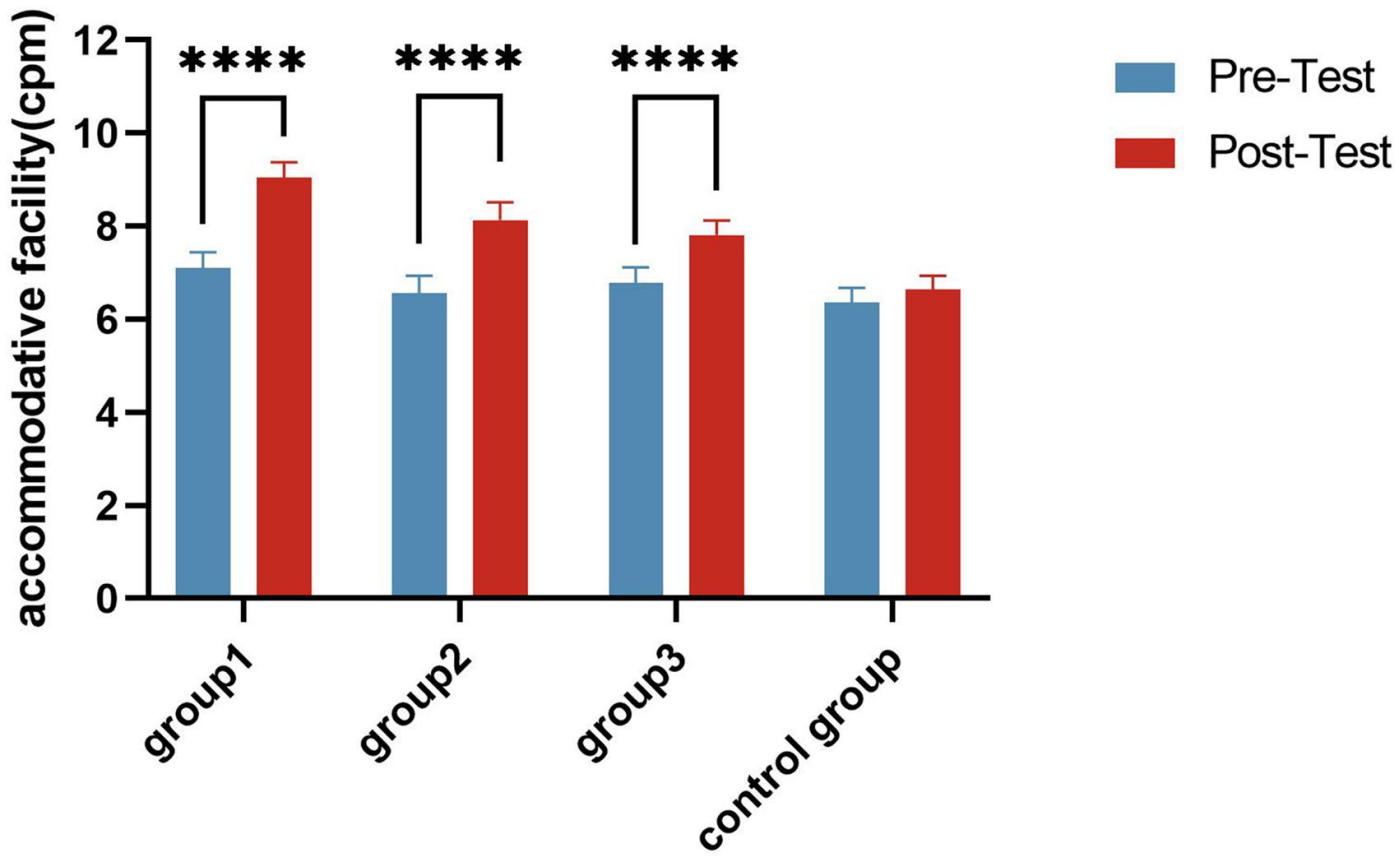
Accommodative facility change trend in groups. ****p ≤ 0.0001.

**Figure 6:**
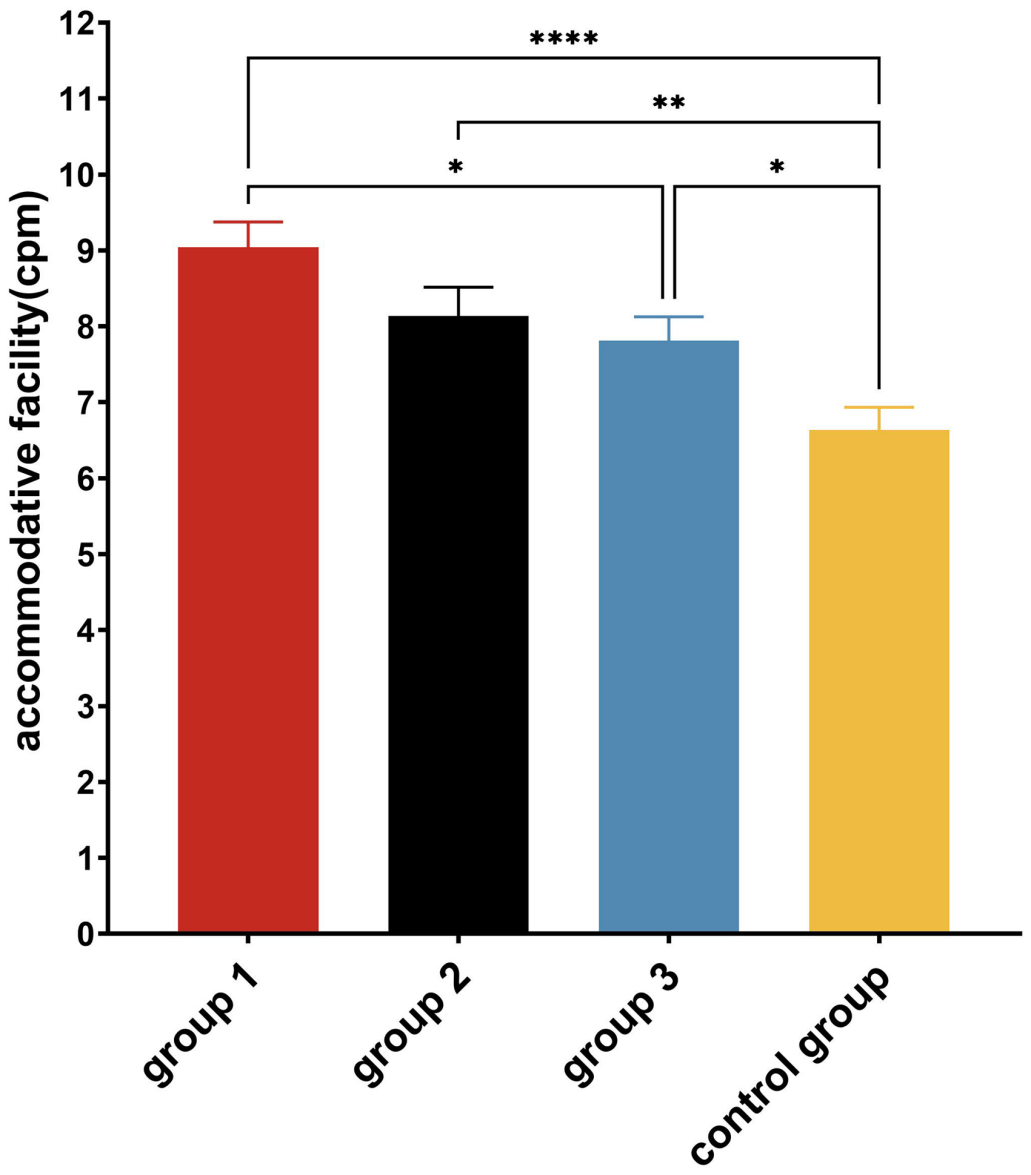
*Post hoc* multiple comparisons of accommodative facility in each group after the experiment. *p ≤ 0.05; **p ≤ 0.01; ****p ≤ 0.0001.

A paired-sample *t*-test revealed that accommodative facilities significantly increased in all experimental groups (*p* < 0.01) after the intervention (Figure 5). A one-way ANOVA showed that there were significant differences in accommodative facilities among the four groups (*F* = 8.533, *p* < 0.001, *η*^2^ = 0.146) (Table 4). The *post hoc* tests showed that accommodative facilities were significantly better in all experimental groups than in the Control Group; Group 1 accommodative facility were significantly better than Group 3 (*p* < 0.05) (Figure 6).

**Table 4:** Comparison of accommodative facility between groups.

Group	Pre-test	Post-test	*t*/*(I-J)*	*P*	Cohen’s *d*
Group 1	7.095 ± 2.140	9.041 ± 2.032	−6.196	0.000	1.019
Group 2	6.550 ± 2.449	8.137 ± 2.391	−4.846	0.000	0.766
Group 3	6.775 ± 2.178	7.813 ± 1.983	−8.084	0.000	1.278
Control group	6.351 ± 1.996	6.635 ± 1.817	−1.796	0.081	0.295
*F*	0.782	8.533			
*P*	0.506	0.000			
*η* ^2^	0.015	0.146			
*Post hoc*		Group 1 > Control group	2.405	0.000	1.161
		Group 2 > Control group	1.502	0.002	0.725
		Group 3 > Control group	1.177	0.014	0.568
		Group 1 > group 3	1.228	0.010	0.593

### 3.3 Comparison of UDVA between groups

**Figure 7:**
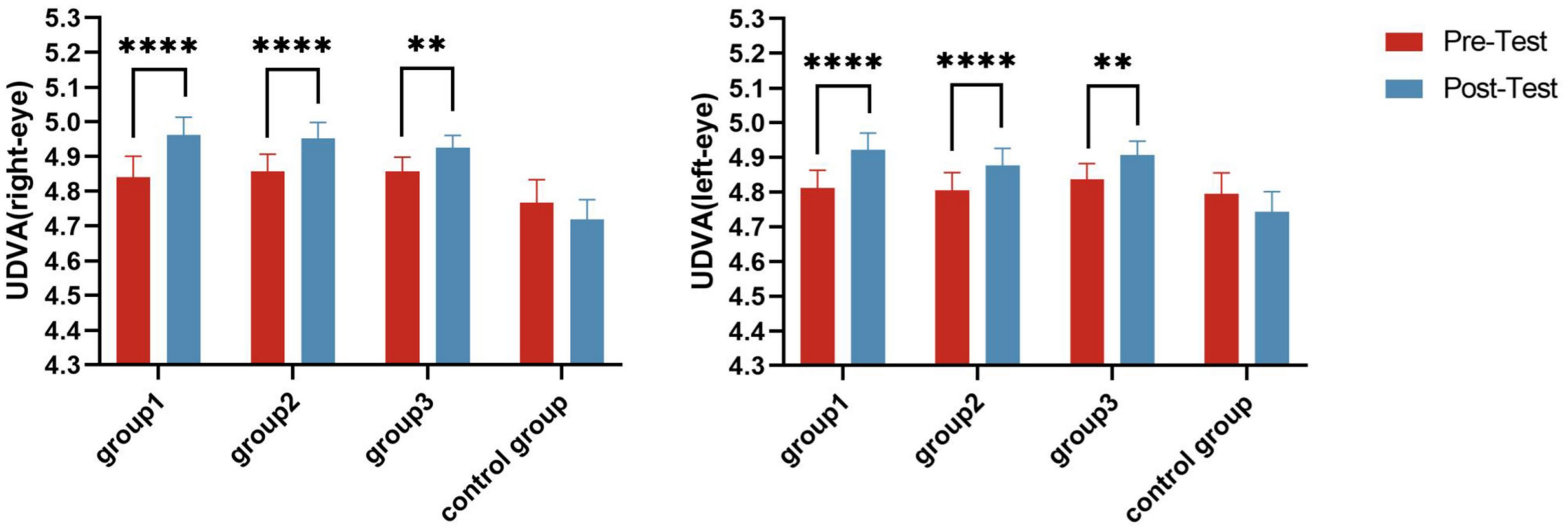
Uncorrected distance visual acuity (UDVA) change trend in groups. **p ≤ 0.01; ****p ≤ 0.0001.

**Figure 8:**
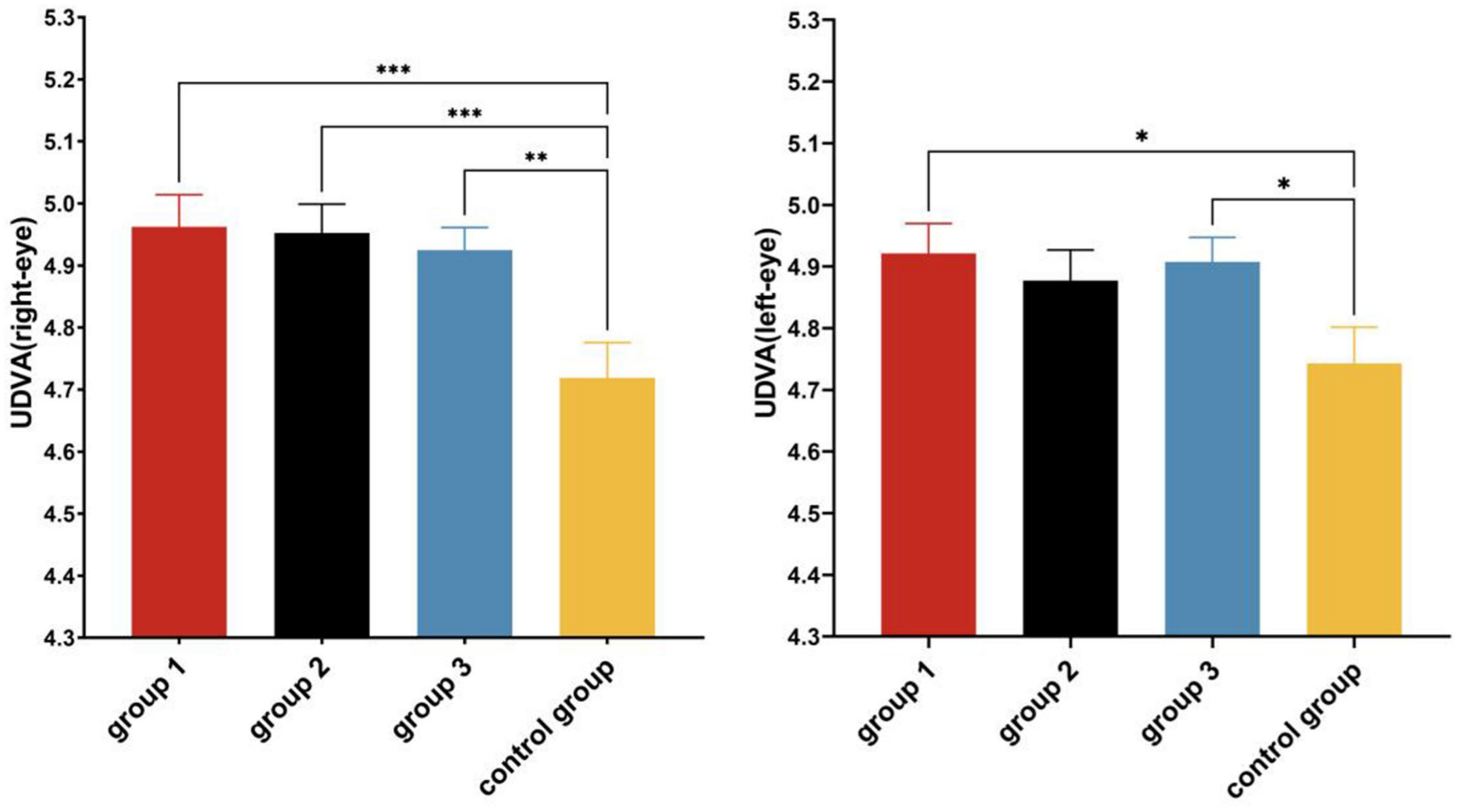
*Post hoc* multiple comparisons of UDVA in each group after the experiment. *p ≤ 0.05; **p ≤ 0.01; ***p ≤ 0.001.

A paired-samples *t*-test showed that after the intervention, the left and right UDVA significantly improved (*p* < 0.05) in all experimental groups (Figure 7). A one-way ANOVA showed that there were significant differences in right-eye UDVA (*F* = 5.550, *p* = 0.001, *η*^2^ = 0.100) and left-eye UDVA (*F* = 2.667, *p* = 0.050, *η*^2^ = 0.051) among the four groups (Tables 5, 6). The post-hoc multiple comparisons showed that Group 1 and Group 3 had significantly better left-eye UDVA than the Control Group, and the right-eye UDVA of each experimental group was significantly better than that of the Control Group (*p* < 0.05) (Figure 8).

**Table 5:** Comparison of right-eye UDVA between groups.

Group	Pre-test	Post-test	*t*/*(I-J)*	*P*	Cohen’s *d*
Group 1	4.841 ± 0.368	4.962 ± 0.316	−7.175	0.000	1.180
Group 2	4.857 ± 0.319	4.952 ± 0.294	−4.554	0.000	0.720
Group 3	4.857 ± 0.262	4.925 ± 0.230	−3.043	0.004	0.481
Control group	4.768 ± 0.400	4.719 ± 0.346	1.901	0.065	0.313
*F*	0.603	5.550			
*P*	0.614	0.001			
*η* ^2^	0.012	0.100			
*Post hoc*		Group 1 > Control group	0.243	0.001	0.816
		Group 2 > Control group	0.234	0.001	0.783
		Group 3 > Control group	0.206	0.003	0.691

**Table 6:** Comparison of left-eye UDVA between groups.

Group	Pre-test	Post-test	*t*/*(I-J)*	*P*	Cohen’s *d*
Group 1	4.811 ± 0.317	4.922 ± 0.294	−6.273	0.000	1.031
Group 2	4.805 ± 0.323	4.877 ± 0.313	−4.529	0.000	0.716
Group 3	4.837 ± 0.287	4.907 ± 0.253	−3.343	0.002	0.529
Control group	4.795 ± 0.370	4.743 ± 0.356	2.006	0.052	0.330
*F*	0.124	2.667			
*P*	0.946	0.050			
*η* ^2^	0.002	0.051			
*Post hoc*		Group 1 > Control group	0.178	0.013	0.584
		Group 3 > Control group	0.164	0.020	0.538

### 3.4 Comparison of imagery ability between groups

A paired-samples *t*-test was used to analyze the pre- and post-experimental imagery abilities of the two imagery groups, and the results showed that after the intervention, the general cognitive abilities of Group 1 significantly improved (*p* < 0.05). The cognitive-specific abilities of Group 1 and Group 2 were significantly improved after the intervention (*p* < 0.05). The independent sample t-test showed that there was no significant difference in the cognitive general imagery ability and cognitive specific imagery ability between Group 1 and Group 2 after the intervention (*p* > 0.05) (Table 7).

**Table 7:** Comparison of imagery ability.

Group	Cognitive general imagery ability					Cognitive specific imagery abilities				
	Pre-test	Post-test	*t*	*P*	Cohen’s *d*	Pre-test	Post-test	*t*	*P*	Cohen’s *d*
Group 1	21.838 ± 2.824	22.892 ± 2.481	−2.275	0.029	0.374	22.432 ± 2.744	24.730 ± 2.704	−5.963	0.000	0.980
Group 2	21.050 ± 3.210	21.750 ± 3.855	−1.512	0.139	0.239	23.175 ± 2.650	24.100 ± 2.146	−2.128	0.040	0.336
*t*	1.140	1.532				−1.208	1.136			
*P*	0.258	0.130				0.231	0.260			

## 4 Discussion

This study demonstrated that motor imagery training improved the participants’ KVA, accommodative facility, and UDVA.

### 4.1 Effect of physical activity on vision levels

This study demonstrated that physical activity improved children’s KVA, accommodative facility, and UDVA, which is consistent with previous studies and reaffirms that physical activity combined with additional visual tasks is an effective means of preventing and managing myopia.

Our study not only examined UDVA but also included KVA and accommodative facility to comprehensively evaluate visual function. Specifically, KVA is the ability to recognize the details of objects moving backward and forward toward the eye, and relies on the ciliary muscles (Cutler and Ley, 1963). Accommodative facility reflects the flexibility of the ciliary muscle in contracting and relaxing. KVA, accommodative facility, and UDVA are closely related to the ciliary muscle. Improvements in all three indicators suggest that physical activity may contribute to better visual health by enhancing ciliary muscle function.

Previous studies have been controversial about the mechanism by which physical activities play a role in myopia prevention and control. There is a general consensus that outdoor factors (e.g., light exposure) are the main reason why physical activity can prevent myopia rather than the physical activity itself, but this study believes that the characteristics of the physical activity itself are also worthy of attention in the prevention of myopia (Wang et al., 2022). During physical activities, children’s eyes frequently shift focus between near and far objects. This frequent accommodation process will promote the ciliary muscle to contract and relax alternately, thereby enhancing its accommodation ability and preventing the occurrence of false myopia and the development of true myopia, which is of positive significance for maintaining children’s eye health.

### 4.2 Effect of motor imagery training on visual acuity and imagery abilities

After 16 weeks of intervention, the children in both motor imagery groups showed improvements in KVA, UDVA, accommodative facility, and cognitive-specific imagery abilities. These results confirm that children can enhance both their imagery abilities and visual acuity through imagery training, even in the absence of external stimuli.

Based on the improved KVA and accommodative facility of the children in the motor imagery intervention groups, it is possible that imagining near and far movements of a visual target may engage visual accommodation mechanisms, potentially including subtle ciliary muscle activity. However, this hypothesis remains speculative. The formulation of this hypothesis is primarily grounded in established theories of ciliary muscle function and motor imagery.

Abnormalities in the structure, innervation, and function of the ciliary muscle are closely related to the onset and progression of myopia (Dai et al., 2021; Gilmartin and Hogan, 1985; Zheng et al., 2011). According to the theory of accommodation, the adjustment of the human eye is primarily completed by the ciliary muscles. With an increase in adjustment, the ciliary muscle moves forward and inward, and the outer, middle, and inner muscle fibers of the ciliary muscle undergo configuration changes, with thickening of the anterior portion and thinning of the posterior portion, which affects the tension of the suspensory ligament and ultimately causes curvature of the lens (Lossing et al., 2012; Richdale et al., 2013; Wagner et al., 2019). This complex process indicates that the ciliary muscle possesses unique structural features, including many ultrastructural and histochemical characteristics of fast-twitch striated muscle (Lv et al., 2020). The ciliary muscle is a unique smooth muscle that exhibits certain characteristics of striated muscle and is innervated by both the sympathetic and parasympathetic nervous systems.

People can autonomously mobilize their mental imagery. Psychoneuromuscular Theory suggests that when people actively imagine a certain scene or behavior, the relevant neurons will be activated, causing neural excitation, which will be transmitted via efferent nerves to the relevant muscles, and the relevant muscles will produce subtle innervation activities. Motor imagery training has also been shown to induce physiological changes in heart rate, respiratory rate, skin blood flow, and skin temperature, suggesting that imagery can elicit a series of responses from muscles and organs under the control of the autonomic nervous system, comprising sympathetic and parasympathetic nerves (Collet et al., 2013; Deschaumes-Molinaro et al., 1992; Peixoto Pinto et al., 2017). Furthermore, the functional equivalence hypothesis posits that imagery and perception share common neural substrates in structure, function, and mechanisms, and that perceptual function can be improved through appropriate imagery training. Based on the aforementioned theories, and considering that the ciliary muscle is controlled by the autonomic nervous system and exhibits certain characteristics of striated muscle, we the following hypothesis and inference: imagery training designed in accordance with the principles of ciliary muscle accommodation may activate the ciliary muscle, eliciting responses during imagery that resemble those occurring during actual near and far vision, thereby improving vision. However, this inference remains to be verified by more direct physiological evidence in future studies.

Moreover, although direct evidence linking motor imagery to ciliary muscle activation remains limited, some studies provide indirect support. Several studies have demonstrated that the eye movements that occur while imagining an object are almost identical to those that occur while perceiving an object. Laeng and Teodorescu (2002) asked participants to imagine a recently viewed picture and found that their eye-scanning paths during the imagery reproduced the perception of the same visual scene. Rozado et al. (2019) showed that the pupil not only responds automatically to light from physical stimuli, but also changes its diameter by imagining the intensity of the light. In addition, other studies have found that when objects are presented at different distances (near or far) and different sizes (large or small), the pupil diameter and the convergence of the eye will also produce corresponding changes (Sulutvedt et al., 2018). This suggests that even if the object, image, or light source is not present in front of the eye, the muscles of the eye can be activated by imagery, and the pupil can be activated by a pattern of activity similar to that seen in physical execution. Given that the medial rectus muscle, the pupillary sphincter, and the ciliary muscle are co-regulated and jointly innervated by the oculomotor nerve, it is reasonable to infer that the ciliary muscle may also be activated during motor imagery. This inference remains to be further verified.

### 4.3 Differences in the effects of intervention methods on vision levels

Based on the results of multiple comparisons, the motor imagery training for target visual movements showed advantages in improving children’s visual acuity levels. Perhaps for children, including a greater number of components of the imagery training content increases task complexity, as simultaneously processing motor and visual information elevates cognitive load. Compared to imagery training of physical activity combined with visual tasks, motor imagery training of visual target movement isolates the visual activity of near and far changes from physical activity imposes a lower cognitive load. This may be more conducive for children to focus on changes in the size and clarity of the visual target during the initial stages of representational training. In addition, whereas imagery is a top-down process based on past experience (Li and Li, 2018). Compared with visual perception tasks, top-down processing is more conducive to the development of autonomous regulation consciousness.

## 5 Conclusion and future directions

Based on the theory of the functional equivalence of imagery and neuropsychological and physiological mechanisms, this study designed an intervention program for motor imagery training based on the principle of ciliary muscle regulation and demonstrated that motor imagery training can improve the visual acuity level of children. Thus, it is likely to be a feasible pathway for the prevention and management of myopia in children. In addition, after the COVID-19 pandemic, we must consider how to prevent and manage myopia in children under restricted conditions. At the implementation level, imagery training is not affected by factors such as venue or weather, making it more convenient to operate. The application of motor imagery training can enable children to obtain more opportunities to promote visual health, provide a stronger boost for the prevention and management of myopia through physical activities, and provide new intervention spaces and ideas for the comprehensive prevention and management of myopia in children. Moreover, as a top-down intervention method, it is beneficial for children to transform passive regulation acquired through physical activity into active regulation, which can be implemented in daily life.

## 6 Limitations of the study

This study has several limitations that should be considered when interpreting the findings. First, participants were recruited from a single primary school in Suzhou, China, which may limit the generalizability of the results to other regions, populations, or cultural contexts. Future studies should include more diverse and representative samples to enhance external validity. Second, potential confounding factors such as screen time, outdoor activity, and family history of myopia were not rigorously controlled. Future research should systematically collect and account for these variables through detailed questionnaires to strengthen the accuracy and reliability of the findings. Third, no long-term follow-up assessment was conducted in this study, and the sustainability of the intervention effects remains unclear. Future research should expand the sample size and add long-term follow-ups to further verify the durability of the intervention effects. Fourth, although participants, instructors, and evaluators were blinded to group allocation, the possibility of a Hawthorne effect and potential expectancy effect cannot be completely ruled out. Fifth, in this study, the cluster random sampling method based on classes was adopted, which may have introduced a clustering effect. Lastly, due to equipment constraints, neurophysiological and ciliary muscle data during motor imagery were not collected. Future studies employing advanced techniques such as ERP and OCT could further elucidate the underlying mechanisms of motor imagery interventions on visual function.

## Data Availability

The raw data supporting the conclusions of this article will be made available by the authors, without undue reservation.
