# Supplementary material for: Improving children’s visual health by integrating motor imagery training into physical education classes
Source: Front Psychol. 2025 Jun 9;16:1587481. doi: 10.3389/fpsyg.2025.1587481 (PMC12184536; doi:10.3389/fpsyg.2025.1587481)
Supplement: Supplementary file 1 [file Data_Sheet_1.pdf]

Enhancing Visual Acuity in Children through Motor Imagery Training

154 children aged 9–10 years

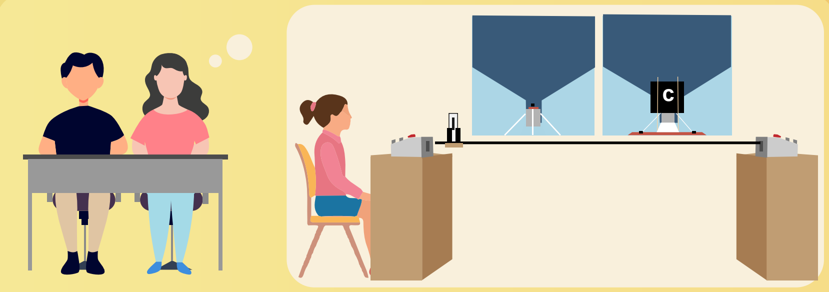

Group 1 Motor imagery:visual targets (n = 37)

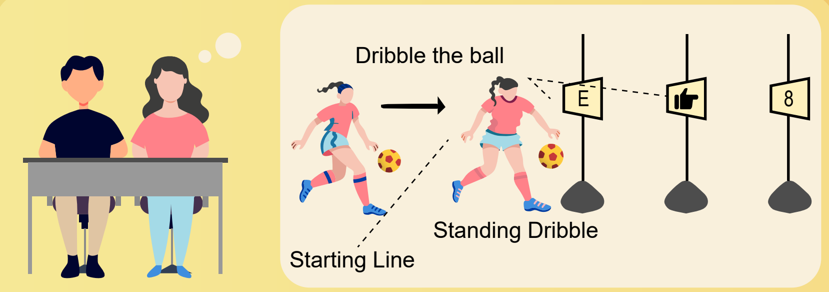

Group 2 Motor imagery:Physical activity+visual tasks (n = 40)

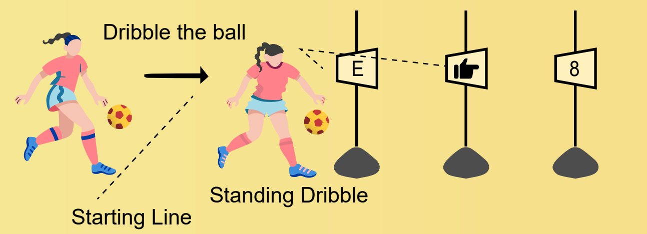

Group 3 : Physical activity+visual tasks (n = 40)

Control group : No intervention (n = 37)

Measurements

Uncorrected distance visual acuity(UDVA)

International Standard Logarithmic Visual Acuity Chart (GB11533-2011)

Kinetic visual acuity (KVA)

Kinetic Visual Acuity Tester

a Landolt ring approaching 50 m away appeared in the apparatus with four notch directions, and a simulated approach speed of 30 km/h

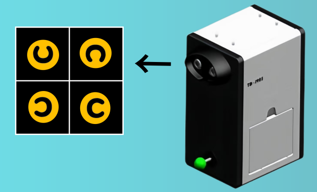

Accommodation facility

Accomodative Flippers

Clulate the number of times alternates between plus and minus lenses within one minute

Motor imagery ability

Sport Imagery Questionnaire

The cognitive general imagery (CG) dimension and the cognitive-specific imagery (CS) dimension subscales

Key findings

After 16-week intervention

↑ kinetic visual acuity, accommodation facility, and uncorrected distance visual acuity improved significantly in all experimental groups($p < 0.05$)

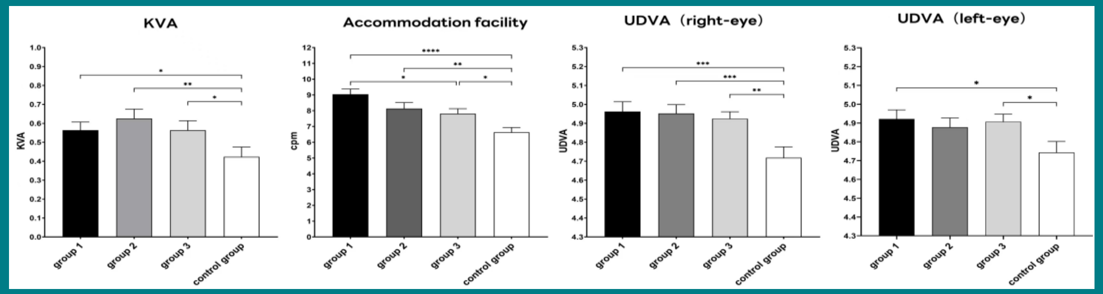

Group 1 showed most improvement in accommodation facility and UDVA

Both motor imagery groups (group 1 and group 2) showed significant cognitive improvements

Motor imagery training improves visual acuity in children by enhancing cognitive and visual skills, potentially aiding in myopia prevention through activation of ciliary muscles
